# 

**Published:** 1891-05

**Authors:** 


### AMERICAN JOURNAL

OF

### DENTAL SCIENCE,

EDITED BY

F. J. S. GORGAS, M. D., D. D. S.
RICHARD GRADY, M. D., D. D. S.

---

VOLUME XXV—Third Series.

---

1891-92.

SURGEON GENL'S OFFICE
141138
LIBRARY

BALTIMORE:
SNOWDEN & COWMAN,

LONDON:
TRUBNER & CO., 60 PATERNOSTER ROW.
1892.

### Index to Volume XXV.---Third Series.

A case Relating to the Influence of Maternal Impressions upon the Fœtus, - - - - 22
A Safe Local Anæsthetic, - - - - 47
Amputation of the Forearm under Cocaine, - 95
Antiseptic Treatment of Wounds, - - - 128
Aneurismal Tumor of the Right Alveolar Process and Vault of the Mouth Treated by Injection, - 170
American Public Health Association, - - - 189
Aristol, - - - - - - 192
A Plea for Conservatism, - - - - 193
American Medical Association, - - - 236
A Gold Clasp, Perfect in Fit, Easily Made, and not Wearing to the Tooth Clasped, - - - - 239
A Correction, - - - - - 284
Aseptinic Acid, - - - - - - 288
Ameriean Academy of Dental Science, - - 327
Antral Abscess as a Result of Alveolar Abscess, - 356
A Case of Cocaine Poisoning, - - - 379
Anti-Extraction, - - - - - 417
A New Society, - - - - - 429
A Safe Local Anæsthetic, - - - - 430
An interesting Reproduction, - - - 432
Antiseptics and Disinfectants used in Dental Surgery, 455
Alveolotomy, - - - - - - 469
A new Styptic, - - - - - 476
A Case in Practice, - - - - - 479
A Convenient Method of Adding New Teeth to Old Plates, 480
Antiseptics and Disinfectants used in Dental Surgery, 481
A Word to Students on the Importance of Mechanical Dentistry, - - - - - 512
A Student of Pharmacy, - - - - 526
American Academy of Dental Science, - - 573
American Dental Society of Europe, - - - 574
Acidity of the Stomach, - - - - 576

Bleeding After Extraction, - - - - 41
Bones Ejected from Her Body, - - - 190
Bacteria of the Human Mouth, - - - - 261
Baltimore College of Dental Surgery, - - 570
Chemistry, Physiology and Pathology of Nitrous Oxide—
Practical Points, with Comments, - - - 27
Central Dental Association of Northern New Jersey.—
Meeting of Committees on Dental Patents, - 40
Chicago Dental Society, - - - - 41
Compound Fracture of the Alveolus and Maxillary, 70
Creolin, - - - - - - 112
Covering Gold Crowns with Glass. - - - 238
Caries, - - - - - - 271
Corrosive Sublimate as a Disinfectant against the Staphylococcus Pyogenes Aureus, - - - 371
Cocaine, - - - - - - 374
Capping Pulps, - - - - - 381
Clinical Nights at the Societies, - - - 425
Cleansing the Hands, - - - - - 430
Chromic Acid in Syphilitic Affections of the Mouth, 474
Concentration as a Principle of Success, Or "One Thing I Do," - - - - - - - 529
Cincinnati College of Medicine and Surgery, Dental Department, - - - - - - 573
Dark Joints, - - - - - - 48
Dr. Edward Maynard, - - - - - 86
Dr. Henry R. Shine, - - - - - 93
Discussion on Anæsthetics in the Therapeutic Section of the British Medical Association, at the Annual General Meeting at Bournemouth, 1891, - 104
Dr. Slich's Discovery, - - - - - 139
Diseases of the Oral Mucous Membrane, - - 212
Disease of the Antrum. - - - - 227
Dermatol, - - - - - - 287
Diseases of the Human Body which have been traced to the action of Mouth-Bacteria, - - - 311
Dentistry in France and America, - - - 330
Dental Medicine, - - - - - 377
Death from Tooth Sepsis, - - - - - 428

Dark Spots on the Teeth, - - - - - 429
Dental Medicine, - - - - - - 472
Dislocations—Fractures of the Jaw, - - - 494
Dentine, - - - - - - - 506
Death from Cocaine Injection, - - - 527
Diseases of the Oral Mucous Membrane, - - 553
Dentists and the Census Bill, - - - - 566
Effects of Sanguinary Deposits on the Teeth, - 20
Extracting Teeth, - - - - - 33
Electricity in Extraction, - - - - 48
Examining Boards vs. Dental Colleges, - - 337
Eruption of the Deciduous Teeth, and General Symptoms, 427
Enlargement of the Cervical Glands, Due to the Impaction of Lower Left Wisdom Tooth, - - - 478
Electricity in Tooth Extraction, - - - 523
Eucalypto Resorcin, - - - - - 526
Finishing the Margins of Teeth, - - - 324
Fitting Bands, - - - - - - 382
Flowers of the Red Rose for Chronic Diarrhœa, - 428
Gold as a Material for Filling Teeth, - - - 368
Gorgas Dental Medicine, Fourth Edition, - - 384
How to Keep Needles and Small Instruments from Rusting, - - - - - - 44
How to Loosen Glass Stoppers, - - - 45
How to Get a Tin Cast, - - - - - 191
Happy Thought, - - - - - - 336
How Gas Cylinders are Made, - - - - 556
Illinois State Dental Society, - - - 41
Iodine in the Treatment of Warts, - - - 47
Infantile Paralysis, - - - - - - 65
Is Dentistry a Dangerous Profession? - - - 241
Insanity following Extraction of Nineteen Teeth, - 285
Is Dentistry a Dangerous Profession, - - 328
James William White, M.D., D.D.S., A.M , - 75
Keep Clean, - - - - - - - 384
Kentucky State Dental Association, - - 575
La Grippe—Origin, History, Treatment, - - 303
Maladies Associated with and Dependent upon Affections of the Teeth, - - - - - - 1

Monthly Meeting of the American Academy of Dental Science, - - - - - - 74
Manipulative Treatment and Dental Pathology, - 141
Music in the Treatment of Nervous Diseases, - 143
Method of Attaching Teeth to Gold Plates by means of Vulcanite, - - - - - 169
Missouri State Dental Association, - - - 188
Maryland State Dental Association, - - - 190
Maryland State Dental Association, - - 285
Maryland State Dental Association, - - - 328
Making an Artificial Set of Teeth, - - - 332
Min..tes of the Twenty third Annual Session of the Southern Dental Association, - - - 385
Mortality of the Human Race, - - - 432
Minutes of the Twenty-third Annual Session of the Southern Dental Association, - -- - 433
National Association of Dental Faculties, - - 175
National Association of Dental Examiners, - 184
Notes on the Anatomy, Physiology and Pathological Significance of Pain and other Nervous Phenomena in Relation to Dental Disease, - - - 219
Notes on a Case of Alveolar Abscess, Complicated with Repeated and Profuse Hæmorrhage and Fracture of Inferior Maxilla, - - - - 231
No More Artificial Teeth, - - - - - 286
National Association of Dental Examiners, - 575
Notes on a Case of Fracture of the Superior Maxillæ, 564
Observations on Bromide of Ethyl in Dental Surgery, 60
Observations on the Decay of the Teeth of Young Mothers, - - - - - - 239
On Some Minor Surgical Operations Performed in or about the Mouth, - - - - 289
Origin of Pus, - - - - - - 410
Obtunding the Bite, - - - - 431
One Step in Advance, - - - - - 503
Phosphate Fillings, - - - - - - 48
Philosophy of Nutrition, with its Legal Aspect, - 136
Permanent Antiseptic Irrigation, - - - 139
Prophylaxis in the Field of the Dental Surgeon, - 163

Physiological Action of Obtundents, - - 203
Pental ($C_5H_{10}$) as an Anæsthetic, - - - 404
Post Graduate Dental Association, - - - 421
Pyoktanin as an Antiseptic, - - - - 426
Profuse Hemorrhage, - - - - 432
Peroxide of Hydrogen as a Therapeutical and Bleaching Agent, - - - - - - . 459
Phenocoll Hydrochlorate, - - - - 477
Plaster of Paris Formula, - - - - 528
Post-Graduate Study, - - - - 541
Resuscitation in Threatened Death from Chloroform, - 34
Risks of Cocaine Injections, - - - 46
Simple Treatment of Ingrown Toe-nail, - - 46
Sensitive Dentine, - - - - - 96
Salol, - - - - - - - - 118
Safety of Hypnotism as a Remedy, - - - 134
Sterilization of Dental Instruments, - - - 209
Samuel Cartwright, F. R. C. S., - - - 236
Saving a Nerve, - - - - - 334
The Education of Physicians and Dentists, - 31
The American Dental Society of Europe, - - 36
The American Dental Association, - - - 39
The Detection of Abscess, - - - - 43
Twenty-third Annual Session of the Georgia State Dental Society, - - - - - - - 49
The Death of Dr. Edward Maynard, - - - 75
The Retention or Removal of the Sixth-Year Molar—Which? - - - - - - - 97
The Rational Use of Vulcanite Rubber, - - 124
The Use of Cocaine as an Anæsthetic, - - 132
Trigeminal Neuralgia and Iodide of Potassium, - 144
The Physiology and Pathology of the Second Dentition, 145
The Twenty-fourth Annual Meeting of the American Academy of Dental Science, - - - 189
The United States Supreme Court Decision in "Tooth Crowns," - - - - - - 240
Twenty-third Annual Session of the Georgia State Dental Society, - - - - - - 251
The Care of the Deciduous Teeth, - - - 277

Third Dentition, - - - - - - 283
To Sterilize Instruments without Dulling Them, - 287
The Care of Children's Teeth, - - - 319
The Missing or Suppressed Teeth in Man. Inaugural Address, - - - - - - - 362
The Physiology of Digestion in Infants, - - 383
The Maryland Dental Law, - - - - 421
Trigeminal Neuralgia and Iodide of Potassium, - 429
Thirty six Teeth in a Set, - - - - 430
Temporary Sets, - - - - - 431
The Human Breath, - - - - - 431
Tumenol, - - - - - - - 472
The Maryland Dental Law, - - - - 514
The Maryland State Dental Association, - - 522
Two Collateral Cases, - - - - - 525
The Implantation of Artificial Teeth, - - 527
The Didactic Method in Dental Teaching, - - 548
Treatment and Destruction of the Pulp, - - 560
The Texas Dental Law, - - - - 568
To Students, - - - - - - 575
Ulcers and Gangrenous Wounds, - - - 480
University of Maryland—Dental Department, - - 572
William F. Wegge, M. D., D. D. S , - - 74
William H. Atkinson, M. D., D. D. S., - - 91
When the Brain is Idle, - - - - 288
Was Cocaine the Cause of this Death? - - 335

### Editorial, Etc.

### THE AMERICAN DENTAL SOCIETY OF EUROPE.

The American Dental Society of Europe will hold its 17th meeting at Heidelberg on the Neckar, in the Schloss Hotel, August 3d, 4 and 5th, 1891.

The members of the Executive Committee and the Secretary of the Society having met in Heidelberg on the 8th of February, 1891, and having made the preliminary arrangements for the coming 17th meeting on Monday, Tuesday and Wednesday, the 3d, 4th and 5th of August, now desire to prepare a full programme for the meeting and to this end request an immediate response from all the members to the following questions:

1. Do you expect and intend to be present at the meeting or not?

2. What do you propose to contribute to the interest of the meeting and in what department?

3. If you are to read a paper, give the title in full and the name of your colleague, who is to open the discussion on the same. (See note below.)

4. Please notify the Hotel Proprietor a few days beforehand of the day of your arrival, as it will be the height of the season, and rooms must be engaged beforehand.

5. Please send the Secretary your name in full and exact address, with the replies to the above questions.

After a careful inspection of the town of Heidelberg, the beautifully situated Schloss Hotel, on the hill above the town, and overlooking the valley and the magnificent ruins of the castle, was selected by the Committee for the place of meeting and special terms secured for the accommodation of from thirty to fifty members and visitors. Those remaining 4 days will secure pension rates of from 8 to 9 marks a day all included, for shorter stay 11 to 12 marks per day.

The programme of the meeting which will be issued early in June, will be arranged to combine excursions and other pleasant recreations with the usual work of the Society, and members will be able to visit and inspect many points of interest about the ruins, the valley, the town and the University during the time of the meeting; and many will find the situation so attractive as to lead them to prolong their stay in this charming place.

The Society will consider the subject of illuminating the ruins of the castle, at night, the cost ranging from 40 to 400 marks.

Thursday morning, the 6th, a party will be formed to visit Frankfort, where the electrical exhibition will be held this year, and the party will enjoy special facilities for inspecting the medical and dental departments.

The Frankfort *rendez vous* will be the dental clinic of Dr. Adams, Burgerstrasse 7, five minutes walk from Union Station, built and established by the Baroness Louise de Rotschild. This is a model institution of this kind elaborately fitted up with all the modern conveniences, and patients may be had for any clinical demonstrations, which members may wish to give, by notifying Dr. Adams in time.

Members of the Society who intend to present papers should make arrangements with some other member to open the discussion on the subject under consideration, and if possible send the paper or a synopsis of it to the said member that he may be able to present his matured thoughts on the subject and intelligently start the discussion that the contents may have due consideration from the Society.

If papers are received by the Secretary in Basle in proper form, by June 1st, he will undertake to have them printed in pamphlet form for distribution at the meeting should the committee decide that their contents are of sufficient general interest to warrant the undertaking.

If notice is given by members who intend to demonstrate or operate clinically, in Heidelberg, arrangements will be made with some dental depot for securing chairs and engines, etc., but smaller instruments must be furnished by the operators.

The committee hopes that members will promise only what they are reasonably sure of being able to present, that other members who attend the meeting may not be disappointed in their expectations.

It has often occurred that members have allowed their names to go out on the programme as intending to present a paper who have not even attended the meeting. Only such papers will be mentioned on the programme as the committee is reasonably sure of.

The proceedings will either be printed by the Society or under special arrangements with some leading Dental Journal, as may be decided at the meeting. Members who intend to present valuable matter should have it in such shape as will make it immediately available for publication.

It will also be desirable to have the remarks of those opening discussions and others participating, written out and handed to the Secretary after the meeting, as a reporter may not be available.

OFFICERS FOR 1891:

President, Dr. Wm. R. Patton, Cologne; Vice-President, Dr. I. B. Davenport, Paris; Treasurer, Dr. C. H. Adams, Frankfort a M.; Secretary, Dr. Lyman C. Bryan, Basel, Switz.; Executive Committee, Drs. Patton, Wetzel, and Adams; Membership Committee, Drs. Davenport, Jenkins and Miller.

---

### THE AMERICAN DENTAL ASSOCIATION.

CHICAGO, May 25th, 1891.

*Editor of American Journal of Dental Science:*

### CENTRAL DENTAL ASSOCIATION OF NORTHERN NEW JERSEY.—MEETING OF COMMITTEES ON DENTAL PATENTS.

There will be a meeting held at the Town Hall, Saratoga, Monday evening, August 3rd, at eight o'clock, of all the committees appointed by the different dental societies all over the United States for the purpose of organizing and taking some action toward the prevention of further issuing of patents upon operations in the mouth. It is hoped that every society will see to it that they are represented at this meeting. Remember it will be held the Monday evening preceding the meeting of the American Dental Association.

S. C. G. WATKINS, *Chairman.* } For the New
W. L. FISH, *Secretary.* } Jersey Com.

### To Readers and Correspondents.

Communications intended for publication, and exchange journals and papers, should be addressed to the Editor of The American Journal of Dental Science, No. 845 N. Eutaw St. Baltimore, Md. All letters relating to the business of the Journal, or containing cash remittances, to be directed to Messrs. Snowden & Cowman, No. 9 W. Fayette Street, Baltimore, Md. Articles for publication should be received by the Editor at least two weeks previous to the first of the month on which the number in which it is designed for them to appear, is issued.

### CONTENTS:

### EDITORIAL.

The American Dental Society of Europe. - - - - 36

The American Dental Association. - - - - - 39

Central Dental Association of Northern New Jersey.—Meeting of Committees on Dental Patents. - - - - - 40

Illinois State Dental Society. - - - - - 41

Chicago Dental Society. - - - - - - - - 41

### MONTHLY SUMMARY.

Bleeding After Extraction. - - - - - - 41

The Detection of Abscess. - - - - - - 43

How to Keep Needles and Small Instruments from Rusting. - 44

How to Loosen Glass Stoppers. - - - - - 45

Risks of Cocaine Injections. - - - - - - 46

Simple Treatment of Ingrown Toenail. - - - - 46

Iodine in the Treatment of Warts. - - - - - 47

A Safe Local Anæsthetic. - - - - - - 47

Electricity in Extraction. - - - - - - - 48

Phosphate Fillings. - - - - - - - 48

Dark Joints. - - - - - - - - - 48

☞ The American Journal of Dental Science will contain fifty pages of reading matter in each number, making a volume of about Six Hundred pages,

EXCLUSIVE OF ADVERTISEMENTS.

